# High mortality among tuberculosis patients on treatment in Nigeria: a retrospective cohort study

**DOI:** 10.1186/s12879-017-2249-4

**Published:** 2017-02-23

**Authors:** Aishatu L. Adamu, Muktar A. Gadanya, Isa S. Abubakar, Abubakar M. Jibo, Musa M. Bello, Auwalu U. Gajida, Musa M. Babashani, Ibrahim Abubakar

**Affiliations:** 1 Department of Community Medicine, College of Health Sciences, Bayero University Kano, Kano, Nigeria; 2 Department of Community Medicine, Aminu Kano Teaching Hospital, Kano, Nigeria; 3 Department of Medicine, College of Health Sciences, Bayero University Kano, Kano, Nigeria; 4 Department of Medicine, Aminu Kano Teaching Hospital, Kano, Nigeria; 5 Institute for Global Health, University College London, London, UK

**Keywords:** Tuberculosis, Mortality, Risk factors, Adults, Nigeria, Retrospective cohort

## Abstract

**Background:**

Tuberculosis (TB) remains a leading cause of death in much of sub-Saharan Africa despite available effective treatment. Prompt initiation of TB treatment and access to antiretroviral therapy (ART) remains vital to the success of TB control. We assessed time to mortality after treatment onset using data from a large treatment centre in Nigeria.

**Methods:**

We analysed a retrospective cohort of TB patients that commenced treatment between January 2010 and December 2014 in Aminu Kano Teaching Hospital. We estimated mortality rates per person-months at risk (pm). Cox proportional hazards model was used to determine risk factors for mortality.

**Results:**

Among 1,424 patients with a median age of 36.6 years, 237 patients (16.6%) died after commencing TB treatment giving a mortality rate of 3.68 per 100 pm of treatment in this cohort. Most deaths occurred soon after treatment onset with a mortality rate of 37.6 per 100 pm in the 1^st^ week of treatment. Risk factors for death were being HIV-positive but not on anti-retroviral treatment (ART) (aHR 1.39(1 · 04–1 · 85)), residence outside the city (aHR 3 · 18(2.28–4.45)), previous TB treatment (aHR 3.48(2.54–4.77)), no microbiological confirmation (aHR 4.96(2.69–9.17)), having both pulmonary and extra-pulmonary TB (aHR 1.45(1.03–2.02), and referral from a non-programme linked clinic/centre (aHR 3.02(2.01–4.53)).

**Conclusions:**

We attribute early deaths in this relatively young cohort to delay in diagnosis and treatment of TB, inadequate treatment of drug-resistant TB, and poor ART access. Considerable expansion and improvement in quality of diagnosis and treatment services for TB and HIV are needed to achieve the sustainable development goal of reducing TB deaths by 95% by 2035.

## Background

Despite effective treatment being available for over half a century, tuberculosis (TB) remains a leading cause of death worldwide [1, 2]. TB deaths have consistently been used as targets and indicators to measure progress towards control [3, 4]. An estimated 1 · 5 million individuals died of TB in 2014, disproportionately affecting countries in sub-Saharan Africa and South-East Asia [5]. In high burden settings, contributory factors include HIV infection (0.4 million deaths), increasing levels of drug resistance and co-morbidities such as diabetes, and social deprivation including homelessness, further amplified by outdated diagnostics, and treatment [6–8].

Recent evidence suggests that TB prevalence and mortality have been under-estimated in many high-burden countries with revised estimates from Nigeria changing global figures in 2013 [9]. Nigeria, the most populous country in Africa, has an estimated population of over 170 million people. Among the identified 22 high burden countries, Nigeria had the highest death rates in HIV-negative people in 2014 (97 per 100,000) and a high HIV-positive TB death rate (44 per 100,000) [5, 10]. Reports indicate that TB prevalence and deaths have been underestimated and the 2015 TB mortality rates in Nigeria may even be higher than the 1990 estimates [5, 10, 11]. TB deaths after treatment has been initiated remain high and occur within the first few months of treatment [12–16]. Burden of drug-resistant TB is high in Nigeria, with an estimated 29,000 (16 per 100,000) new cases in 2015 [17]. Treatment and microscopy services are provided free in 5,728 treatment centres and 1,765 microscopy sites across the country, though coverage is disproportionately higher in urban areas [18]. Among an estimated 3.4 million people living with HIV in Nigeria, less than 800,000 are on antiretroviral therapy [19].

Although there are studies describing TB treatment outcomes in Nigeria, [20–27] there is paucity of information on mortality in HIV negative and HIV positive cohorts. Additionally, the Boko Haram insurgency in the North-eastern part of the country since 2009, has led to a population of internally displaced persons from communities often with no access to healthcare who may be at a higher risk of disease and death. For instance, a survey of multi-drug resistant TB in North-east states affected by this conflict showed a prevalence of up to 35.7% in most affected state (Borno) [28]. Consequently, in this study we evaluate mortality and factors associated with time to death in a large treatment centre.

## Methods

### Study setting

Kano state in Northern Nigeria is one of the most populous in the country with over 12 million residents and has the third highest number of TB cases notified to the National TB programme [18]. Aminu Kano Teaching Hospital (AKTH), is a large federal government run university hospital, established in 1988, which caters for populations from Kano and neighbouring states. It has a turnover of about 400,000 out-patients and over 19,000 in-patient admissions reported in 2013.

The AKTH TB-DOTS clinic provides TB screening, diagnosis and treatment services to both children and adults. Patients enrolled in the DOTS service come from a variety of sources and include suspected and confirmed TB cases, referred from other clinics within the hospital and other hospitals including private health facilities. Other services provided are HIV counselling and testing, contact tracing, and provision of isoniazid-prophylaxis to children in close contact with active TB cases.

Patients diagnosed with TB receive treatment based on the existing national guidelines. Prior to 2014, category 1 treatment (CAT 1) was given to new patients which comprised 2 months of Rifampicin (R), Isoniazid (H), Pyrazinamide (Z) and Ethambutol (E) followed by 6 months of EH (up to 2012) or 4 months of RH (from 2013); and CAT 2 regimen for re-treatment patients, comprised 2 months of RHZE and Streptomycin (S), 1 month of RHZE and 5 months of RHE. However, from 2014, the guideline recommended the same regimen 2RHZE and 4RH for both new and re-treatment cases with the exception of central nervous system TB which requires longer treatment. Treatment is also occasionally extended if final investigations such as sputum smear or X-ray are not available. Guidelines for investigation (including a GeneXpert machine in the TB lab), management and referral of multi-drug resistant TB became available from 2014.

TB Treatment is observed by the health worker in the clinic once weekly during the intensive phase, and once monthly in the continuation phase. Daily supervision is undertaken by a designated guardian.

TB/HIV services are co-located and integrated. Patients with unknown HIV status at enrolment are routinely counselled and screened for HIV. Patients with HIV are followed up in the same clinic. Anti-retroviral treatment (ART) is based on existing National guidelines, and eligibility is based largely on clinical staging of HIV disease and CD4 count. However, for co-infected patients that are newly diagnosed with HIV or those diagnosed with TB before commencement of ART, guidelines recommend commencement of ART irrespective of HIV disease stage or CD4 count. Though only ART status at beginning of TB treatment is usually recorded in the TB registers.

### Study design data sources

Patients 15 years and above enrolled in the clinic for treatment from January 2010 to December 2014 were included in the cohort. Data were collected from a combination of clinic-based sources - patient treatment cards and TB treatment registers. A treatment card is opened for each patient upon registration in the clinic on treatment commencement, and each patient is assigned a unique identification number. Patient and treatment information including treatment outcome are recorded in both.

Information available included: age, gender, residence, sputum smear status, site of disease, HIV status at treatment onset, previous TB treatment, ART status at treatment onset, date of treatment onset, treatment outcome, and date of censor. Age was categorised into the following bands: 15–24, 25–34, 35–44, 45–54 and ≥65 years. HIV/ART status was determined from HIV and ART status at treatment onset and grouped in to HIV-negative, HIV-positive on ART, HIV-positive not on ART and Unknown HIV status. Site of disease was classified in to pulmonary (disease affecting lungs only), extra-pulmonary (disease affecting organs other than the lung) and both (disease affecting the lungs and any other organ) and patients were also group into bacteriologically-confirmed (sputum-smear or culture confirmed) or clinically diagnosed (smear-negative and physician-confirmed through other means). Residential status includes Kano residents (primarily residing within Kano metropolis) and outside Kano.

Subjects entered the cohort on the day treatment commenced and remained in the cohort until any one of the following occurred; i) end of treatment ii) death before end of treatment iii) loss to follow-up from default or unknown treatment outcome status. The time between treatment onset and death was the primary outcome which included all causes based on the WHO 2013 definitions for treatment outcome [29].

### Statistical analysis

We described numbers and proportions for categorical variables and means (with standard deviation, SD) or medians (with interquartile range) for quantitative measures for all patients. To inform the analyses, follow-up time was time from treatment commencement to date of censor which was expanded into person-months (pm) at risk. Mortality rates over time per 100 pm at risk were estimated and plotted by time since treatment commencement. Covariate stratum-specific mortality rates were also determined. Kaplan-Meier analysis and log-rank tests were used to compare survival curves stratified by previous TB treatment and HIV/ART status. To estimate hazard ratios (HR) with corresponding 95% confidence intervals (CI), Cox proportional hazards modelling was fitted and used to determine risk factors for mortality. Covariates that were associated with each outcome measure (adjusting for age) with *p* < 0 · 2 were included in the multivariable analysis. Variables were included in models if they resulted in >10% change-in-estimate or a change in log likelihood with p-value < 0 · 2. Variables that resulted in change in coefficient standard errors of already included variables by >20% were assumed to be collinear and excluded from the model.

All analyses were done using Stata 14 (Stata Corp, College Station, TX, USA).

## Results

**Fig. 1:**
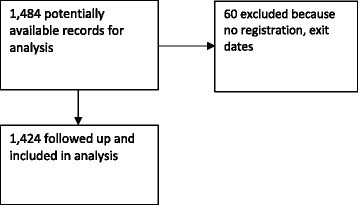
Study participants

Among 1,484 eligible patients, 1424 (96.0%) were followed-up for analysis (Fig. 1). More than half of the patients were men (56.9%) and the mean age was 36.6 ± SD14.4 years. The study cohort characteristics and treatment outcomes are shown in Table 1. About a quarter (342) had reported a history of being previously treated for TB. Out of the 568 (40%) participants that were HIV positive at treatment onset, only 90 (15.8%) were on ART. Over two-thirds (936) of the patients had pulmonary tuberculosis, while 223 (16.1%) had both pulmonary and extra-pulmonary TB. Common extrapulmonary sites of TB included abdomen, spine, CNS, lymph nodes, and pericardium.

**Table 1:** Description of study cohort

Variable	Frequency	Percent
Age group (years)		
15–24	260	19.5
25–34	396	30.0
35–44	335	25.1
45–54	185	13.9
55–64	87	6.5
> 65	72	5.4
Missing	93	
Gender		
Male	81	56.9
Female	614	43.1
Residence		
Within Kano	1,014	71.2
Outside Kano	410	28.8
Referral facility		
DOTS-linked facility	624	45.7
Non DOTS-linked facility	740	54.3
Missing	60	
TB diagnosis		
Bacteriological	468	32.9
Clinical	956	67.1
TB site		
Pulmonary	936	67.4
Extra-pulmonary	229	16.5
Both	223	16.1
Missing	36	
HIV/ART status		
HIV-	683	48.0
HIV+ on ART	90	6.3
HIV+ not on ART	478	33.6
Unknown HIV status	173	12.2
Previous TB treatment		
No	1,082	76.0
Yes	342	24.0
Year of diagnosis		
2010–2011	413	29.0
2012	321	22.5
2013	450	31.6
2014	240	16.9
Treatment outcome		
Successful	745	52.3
Lost to follow-up	366	25.7
Died	237	16.6
Failed	33	2.3
Transferred-out	43	3.0

Characteristics of the 60 patients that were excluded from this analysis are shown in Table 2. Their mean age (SD) was 36.4 ± SD14.0 years, and 27 (45%) were HIV-positive, while eight (13.3%) had unknown HIV status.

**Table 2:** Characteristics of 60 patients excluded from analysis

Variable	Freq (%) *N* = 60
Age group (years)	
15–24	15 (25.0)
25–34	13 (21.7)
35–44	16 (26.7)
45–54	7 (11.7)
55–64	6 (10.0)
> 65	3 (5.0)
Gender	
Female	27 (45.0)
Male	33 (55.0)
Residence	
Within Kano	42 (70.0)
Outside Kano	18 (30.0)
TB diagnosis	
Bacteriological	2 (3.3)
Clinical	58 (96.7)
TB site	
Pulmonary	29 (82.9)
Extra-pulmonary	5 (14.3)
Both	1 (2.9)
HIV/ART status	
HIV-	25 (41.7)
HIV+ on ART	0 (0.0)
HIV+ not on ART	17 (28.3)
Unknown HIV status	8 (13.3)
Previous TB treatment	
No	56 (93.3)
Yes	4 (6.7)

### Crude mortality

During a total follow-up time of 6436.2 person-months in 1,424 patients, 237 participants died.

Overall case fatality was 16.6% and the crude mortality rate was 3.68 (95% CI – 3.24–4.18) per 100 person-months of follow-up. Crude mortality rates (per 100 person-months) across different covariates are shown in Table 3. Mortality was highest in participants aged 45–54 years and those 65 years or more, although the majority of deaths occurred among those aged 25–54 years. Males had a slightly higher crude mortality rate (4.15:95% CI – 3.53–4.87) than females (3.11:95% CI – 2.53–3.83). Nearly 22% of deaths occurred in HIV positive individuals, however, HIV-positive patients who were on ART at treatment onset had the lowest crude mortality rates (1.52:95% CI – 0.72–3.19).

**Table 3:** Mortality rates across explanatory variables

Variable	Number	Number of deaths (%)	Person-months (pm)	Mortality rate per 100 pm (95% CI)
Age group (years)				
15–24	260	25 (9 · 6)	1344 · 0	1 · 86 (1 · 26-2 · 75)
25–34	395	60 (15 · 2)	1874 · 8	3 · 20 (2 · 49-4 · 12)
35–44	334	50 (15 · 0)	1512 · 2	3 · 31 (2 · 51-4 · 36)
45–54	185	57 (30 · 8)	650 · 8	8 · 76 (6 · 76-11 · 36)
55–64	85	14 (16 · 5)	344 · 5	4 · 06 (2 · 41-6 · 86)
> 65	72	22 (30 · 6)	251 · 7	8 · 74 (5 · 76-13 · 27)
Gender				
Male	810	147 (18 · 2)	3544 · 7	4 · 15 (3 · 53-4 · 87)
Female	614	90 (14 · 7)	2891 · 5	3 · 11 (2 · 53-3 · 83)
Residence				
Within Kano	1,014	76 (7 · 5)	5208 · 8	1 · 46 (1 · 17-1 · 83)
Outside Kano	410	161 (39 · 3)	1227 · 4	13 · 12 (11 · 24-15 · 31)
Referral facility				
DOTS-linked facility	624	34 (5 · 5)	2470 · 7	1 · 05 (0 · 75-1 · 47)
Non DOTS-linked facility	740	201 (27 · 2)	2907 · 0	6 · 91 (6 · 02-7 · 94)
TB diagnosis				
Bacteriological	468	13 (2 · 8)	2819 · 1	0 · 46 (0 · 27-0 · 79)
Clinical	956	224 (23 · 4)	3617 · 1	6 · 19 (5 · 43-7 · 05)
TB site				
Pulmonary	936	105 (11 · 2)	4527 · 9	2 · 32 (1 · 92-2 · 81)
Extra-pulmonary	229	36 (15 · 7)	1146 · 3	3 · 14 (2 · 27-4 · 35)
Both	223	95 (42 · 6)	719 · 3	13 · 21 (10 · 80-16 · 15)
HIV/ART status				
HIV-	683	111 (16 · 3)	3271 · 4	3 · 39 (2 · 82-4 · 09)
HIV+ not on ART	478	104 (21 · 8)	1895 · 4	5 · 49 (4 · 53-6 · 65)
HIV+ on ART	90	7 (7 · 8)	460 · 4	1 · 52 (0 · 72-3 · 19)
Unknown HIV status	173	15 (8 · 8)	809 · 0	1 · 85 (1 · 12-3 · 08)
Previous TB treatment				
No	1.082	93 (8 · 6)	5340 · 9	1 · 74 (1 · 42-2 · 13)
Yes	342	144 (42 · 1)	1095 · 3	13 · 15 (11 · 17-15 · 48)
Year of diagnosis				
2010–2011	413	76 (18 · 4)	1721 · 5	4 · 41 (3 · 52-5 · 53)
2012	321	81 (25 · 2)	1319 · 1	6 · 14 (4 · 94-7 · 63)
2013	450	53 (11 · 8)	2195 · 3	2 · 41 (1 · 84-3 · 16)
2014	240	27 (11 · 3)	1200 · 2	2 · 25 (1 · 54-3 · 28)

**Fig. 2:**
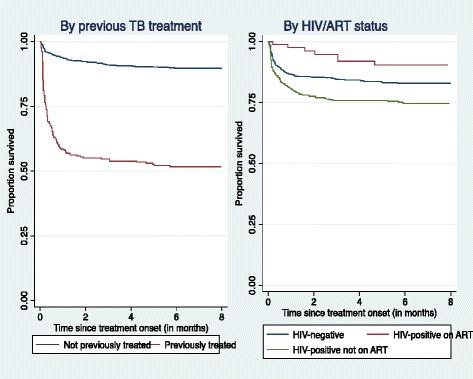
Kaplan-Maier curves showing survival by previous TB treatment and by HIV/ART status among study cohort

Kaplan-Maier survival curves show differences in survival between sub-groups (Fig. 2). When stratified by previous TB treatment, survival was lower for patients with previous TB treatment(*p* < 0.0001). When stratified by HIV/ART status, survival was lowest among HIV-infected but not on ART (*p* = 0.001). Analysis of mortality from time since treatment onset (Table 4) showed that most deaths occurred within the 1^st^ week of treatment, with a crude mortality rate of 37.6 (95% CI – 31.4–44.9) per 100 person-months of treatment. Death rates rapidly declined over treatment period.

**Table 4:** Mortality from time since treatment commencement

Time since treatment onset	Number of deaths	Proportionate mortality (%)	Person-months	Mortality rate per 100 pm (95%CI)	Crude HR (95% CI)	Adjusted HR (95% CI)^a^
1st week	120	50 · 6	319 · 3	37 · 6 (31 · 4-44 · 9)	1	1
2nd week	32	13 · 5	265 · 9	12 · 0 (8 · 5-17 · 0)	0 · 34 (0 · 23-0 · 53)	0 · 49 (0 · 31-0 · 78)
3rd week	23	9 · 7	253 · 2	9 · 1 (6 · 0-13 · 7)	0 · 31 (0 · 19-0 · 49)	0 · 57 (0 · 34-0 · 96)
4th week	13	5 · 5	245 · 9	5 · 3 (3 · 1-9 · 1)	0 · 18 (0 · 10-0 · 32)	0 · 31 (0 · 17-0 · 60)
2nd month	21	8 · 9	936 · 8	2 · 2 (1 · 5-3 · 4)	0 · 08 (0 · 05-0 · 12)	0 · 18 (0 · 10-0 · 30)
3rd month	12	5 · 1	885 · 2	1 · 4 (0 · 8-2 · 4)	0 · 04 (0 · 02-0 · 08)	0 · 13 (0 · 07-0 · 26)
4th month+	16	6 · 7	3530 · 0	0 · 5 (0 · 3-0 · 7)	0 · 01 (0 · 06-0 · 02)	0 · 05 (0 · 03-0 · 08)

^a^Adjusted for age, gender, residence, calendar period, HIV/ART status, previous TB treatment, TB site, referring source, and diagnosis

### Multivariable analysis

After adjusting for age, sex, and other covariates, risk of death was still markedly higher in the 1^st^ week of treatment and declined steadily over treatment period (Table 4). Multivariable analysis (Table 5) showed that, compared to patients aged 15–24 years, those between 45 and 54 years had over twice the risk of death (aHR 2.37: 95% CI – (1.44–3.92)). Risk factors for death were being HIV-positive but not on ART (aHR 1.39:(1.04–1.85)), residence outside the city (aHR 3.18(2.28–4.45)), previous TB treatment (aHR 3.48(2.54–4.77)), no microbiological confirmation (aHR 4.96(2.69–9.17)), having both pulmonary and extra-pulmonary TB (aHR 1.45(1.03–2.02)), and referral from a non-programme linked clinic/centre (aHR 3.02(2.01–4.53)). Calendar period also had a strong effect on risk of death in both univariable and multivariable models, such that patients enrolled at the later periods (2013 and 2014) had lower risks of death compared to those enrolled earlier (2010–2011).

**Table 5:** Univariable and multivariable analyses of risk factors for mortality^a^

Variable	Crude HR (95% CI)	*P* value	Adjusted HR (95% CI)	*p* value
Age group (years)				
15–24	1		1	
25–34	1 · 62 (1 · 01–2 · 58)		1 · 18 (0 · 73–1 · 89)	
35–44	1 · 57 (0 · 97–2 · 53)		1 · 07 (0 · 65–1 · 77)	
45–54	3 · 79 (2 · 37–6 · 07)		2 · 37 (1 · 44–3 · 92)	
55–64	1 · 85 (0 · 96–3 · 57)		1 · 43 (0 · 73–2 · 80)	
> 65	3 · 60 (2 · 03–6 · 38)	<0 · 001	1 · 64 (0 · 90–2 · 99)	0.001
Gender				
Female	1	0 · 06	1	
Male	1 · 28 (0 · 99–1 · 67)		0 · 94 (0 · 71–1 · 25)	0 · 68
Residence				
Within Kano	1		1	
Outside Kano	6. · 74 (5 · 13–8 · 86)	<0 · 001	3 · 18 (2 · 28–4 · 45)	<0 · 001
Referral facility				
DOTS-linked facility	1		1	
Non DOTS-linked facility	5 · 85 (4 · 07–8 · 42)	<0 · 001	3 · 02 (2 · 01–4 · 53)	<0 · 001
TB diagnosis				
Bacteriological	1		1	
Clinical	10 · 72 (6 · 12–18 · 76)	<0 · 001	4 · 96 (2 · 69–9 · 17)	<0 · 001
TB site				
Pulmonary	1		1	
Extra-pulmonary	1 · 46 (1 · 00–2 · 14)		1 · 08 (0 · 72–1 · 63)	
Both	4 · 86 (3 · 68–6 · 42)	<0 · 001	1 · 45 (1 · 03–2 · 02)	0 · 09
HIV/ART status				
HIV-	1		1	
HIV+ on ART	0 · 43 (0 · 21–0 · 92)		1 · 51 (0 · 60–3 · 78)	
HIV+ not on ART	1 · 42 (1 · 09–1 · 86)		1 · 39 (1 · 04–1 · 85)	
Unknown HIV status	0 · 54 (0 · 32–0 · 93)	<0 · 001	0 · 77 (0 · 44–1 · 33)	0 · 05
Previous TB treatment				
No	1		1	
Yes	6 · 43 (4 · 95–8 · 35)	<0 · 001	3 · 48 (2 · 54–4 · 77)	<0 · 001
Year of diagnosis				
2010–2011	1		1	
2012	1 · 17 (0 · 86–1 · 60)		1 · 10 (0 · 78–1 · 56)	
2013	0 · 48 (0 · 36–0 · 68)		0 · 47 (0 · 32–0 · 69)	
2014	0 · 45 (0 · 29–0 · 70)	<0 · 001	0 · 45 (0 · 27–0 · 74)	<0 · 001

^a^Adjusted for all variables in the table

## Discussion

This study has identified a high case fatality rate among a cohort of TB patients on treatment in Nigeria. The majority of deaths in this young cohort occur shortly after treatment onset. We have compared mortality across different risk factors. Factors associated with mortality included HIV infection without ART, residence outside the city, extrapulmonary and pulmonary disease, prior TB treatment, the absence of microbiological confirmation of TB and periods proximal to treatment onset.

Deaths among patients on TB treatment reported from different regions of Nigeria are much lower (<10%) than figures observed in this study (16.6%), except for some that involved rural health facilities (13.1%) [30], tertiary health facility (14.8%) [31], elderly (12.3%) [32] and MDR-TB (15%) [33]. However, following the Boko Haram insurgency, Kano state is home to a large number displaced and vulnerable populations that may also have accounted for high mortality. Case fatality reported from other sub-Saharan Africa ranged between 4 and 9% in HIV-uninfected, lower than our finding and 16–35% among HIV-infected, comparable to our observed 20% in this cohort [34]. Case fatality during treatment from outside Africa were lower, other than in some Asian countries where the cohorts are much older. For example, in a population-wide studies in Taipei Taiwan, case fatality was 17 · 8% for all adults (mean age 65 years) [35] and 25% in another study of elderly persons aged 65 or more years (mean age 80 years) [36], compared to a mean age of 36.6 years in our study. In Singapore, a nationwide study found a case fatality of only 11 · 9%, even though their population was much older and HIV prevalence was very low (0 · 6%) [37]. In India, case fatality was also much lower (6%), even though the age distribution was similar to ours (mean – 36 years) [13] possibly due to a lower HIV prevalence and better access to care. In South Africa, where burden of HIV and drug resistant TB are high, case fatality among platinum miners was 12 · 2% [12].

About half of the deaths occurred within the 1^st^ week of treatment, and over three-quarters within the first month. This is higher than what was obtained in Malawian hospital based study, where 40% of deaths occurred within the 1^st^ month of treatment [38]. Early deaths or clinical worsening after treatment onset have been associated with disease progression from additional co-morbid illnesses, drug toxicity and poor adherence [14, 15, 39–43]. The high death rates observed so soon after treatment onset may be attributable to delays in TB diagnosis and/or initiation of anti-TB and antiretroviral treatment; severe disease; and undiagnosed drug-resistant TB or co-morbidities.

Delays could be in health care-seeking, diagnosis, treatment or a combination of them all [44, 45]. In a review of 45 studies across 17 Asian countries, consultation with a public hospital was associated with lower risk of treatment delay [46]. A systematic review of diagnosis and treatment delays in India showed that use of Government health care providers was a risk factor for patient-related delays while use of private health care providers were associated with health system delays [45]. Reports from sub-Saharan Africa indicate that delay was associated with prior consultation of traditional healers, private or rural public facilities [47–49]. Although sputum smear microscopy is provided to patients free by the National TB programme, additional investigations for diagnosis of TB such as radiographs and histology, and other co-morbidities such as diabetes have to be paid for by the patient and may also contribute to delays and disease progression [50–53]. In a study of platinum miners in South Africa, although high death rates were also observed in the first month of treatment irrespective of HIV/ART status and previous treatment, death rates were still lower than present study. However, the miners were covered by a free comprehensive medical care including HIV care, reducing the likelihood of diagnostic and treatment delays and possibly improving early likelihood of detecting and managing co-existing conditions. The majority of treatment services in Nigeria are provided by lower cadre health workers at the primary care level, with limited capacity for diagnosis of sputum-negative and extra-pulmonary TB. This may have worsened diagnostic delay. Although the literature suggests that access to public hospitals lowers the risk of delay, a large tertiary hospital may represent a different experience within the TB programme due to the extreme of spectrum of disease. It is therefore possible that early deaths in this cohort are reflective of more severe disease

Decrease in mortality rates we observed over calendar period could be reflective of changes in treatment policies which include switch from 8 months (2RHZE/6EH) to 6 months (2RHZE/4RH) treatment regimen; use of same regimen for re-treatment cases; revision of CD4 cell count threshold for ART initiation; and introduction of drug-susceptibility testing. This decrease in mortality could also be as a result of maturity of the DOTS programme within the hospital and country at large, as such delays in diagnosis and treatment may have reduced over time; and detection and management of drug-resistant TB may have improved.

Residing outside Kano city was also associated with increased mortality. Distance from health centre, coexisting untreated or undiagnosed co-morbid chronic illnesses, unavailability of health/treatment centres at place of residence, poor access to ART, and shortage or interrupted supply of drugs can also contribute to deaths [46, 54, 55]. Additionally, rural areas have fewer TB treatment and ART services further decreasing access. Delayed treatment initiation has been shown to be associated with increased periods of infectiousness, disease severity, treatment failure and mortality [56]. Poor treatment outcomes may reflect socio-economic conditions including poor awareness and rural residence which influence access to overall health care [45, 46, 57]. Poor health access may also indicate unrecognised co-morbidities, which can increase risk of death.

About a quarter of the cases were on TB re-treatment, therefore, it is possible that previous failed treatment and drug-resistant TB contributed to the poor outcomes [58, 59]. There were no guidelines or protocol for management of drug resistant TB in the country during the initial period covered by this study, and empirical treatment with 1^st^ line anti-tuberculous agents was recommended. A study in AKTH, Kano reported MDR-TB prevalence of 10.6% [60]. Applying this figure to our cohort implies that inadequate treatment of drug-resistant TB may have increased risk of mortality.

Consistent with previous reports, higher mortality risk was observed among HIV-infected patients not on ART, while those who were already on ART when TB treatment was started did not have any excess mortality risk [61]. Though some studies have shown higher mortality risk among TB/HIV co-infected soon after initiating ART, use of ART for at least 6 months has been shown to reduce mortality [62, 63]. Integrated ART at time of TB treatment initiation has been shown to reduce mortality [61, 64–66]. The integration of TB-HIV services in this centre with active clinical screening for TB, could have resulted in early diagnosis and timely TB treatment among patients already enrolled in the HIV clinic [67]. Our findings indicate that only 15.8% of HIV-infected were on ART at the time of TB treatment. This figure however does not take into account patients who may receive ART during TB treatment, as well as duration of ART treatment, CD4 count and HIV disease stage because these were not updated in the clinic registers. This may have resulted in under-estimation of the effect of ART. The observed effect of HIV on risk of death over time was lower than in other studies across sub-Saharan Africa [12, 20, 65, 68].

Misdiagnosis of other diseases, especially among HIV-infected persons as smear-negative or extrapulmonary TB may partly explain the high mortality rates observed in clinically-diagnosed patients [44, 69, 70]. For instance, a study in South Africa found a strong association between certainty of diagnosis and mortality [12]. Though studies have shown that autopsy-confirmed causes of deaths attributed to TB are lower than those clinically diagnosed, autopsies performed on patients whose deaths had been attributed to other causes have also shown a substantial proportion of missed TB cases [12, 65, 71, 72].

The major strengths of this study include the availability of information allowing time to event analysis which is usually lacking from programme-level data. Findings should be interpreted in light of a number of limitations. First, some deaths in this study may not be TB deaths and would be more appropriately grouped as deaths during TB treatment. Thus, we may have over-estimated TB mortality. Conversely, we might have underestimated the overall mortality in this population as many deaths will likely occur before contact with the health system or the TB programme. Second, although, patients or their next of kin are usually contacted on reasons for default, a number of patients who had been lost to follow-up may have died at home [73]. This may have further under-estimated mortality. Additionally, death rates among the 60 patients excluded may be different, and this may have resulted in either under- or over-estimation of mortality in this cohort. Third, our findings are from a single treatment centre, therefore, generalisability may be somewhat limited. However, we believe that our findings represent the experience of many treatment centres within tertiary-level health facilities in the country which tend to see more of severe disease. Fourth, due to the retrospective nature of this study, we could not account for the effect of other co-morbidities such as diabetes on the risk of death as this information is not recorded in the clinic registers.

## Conclusion

The high TB mortality observed in a tertiary health centre may reflect marked delays in diagnosis; poor access to care among vulnerable populations; and unrecognised co-existing morbidities. Data from our high-TB burden low-resource setting illustrates the difficulties related to diagnosis and treatment of smear negative and extrapulmonary TB, low ART coverage, poor access to comprehensive healthcare and the limitations of passive case-finding. TB programmes in Africa have focused on passively detecting and treating smear-positive disease despite evidence of increasing burden of smear-negative TB including HIV-related TB [34]. To attain the End TB strategy’s target of zero deaths and suffering from TB, aggressive strategies need to be employed to actively find and promptly treat all cases; and make TB care more comprehensive in order to recognise and adequately manage other co-morbidities. Wider access to ART among HIV infected persons will also be critical to the TB and HIV control effort. Considering the limitations of the historical nature of this study, future prospective studies should aim to differentiate deaths due to TB from coincidental deaths from other causes to adequately define TB burden; and identify co-morbidities and their contribution to risk of death among patients with TB.

## Abbreviations

AKTH: Aminu Kano Teaching Hospital
ART: Anti-retroviral treatment
CI: Confidence intervals
DOTS: Directly observed treatment short-course
HIV: Human immuno-deficiency virus
HR: Hazard ratio
MDR: Multi-drug resistant
TB: Tuberculosis
WHO: World Health Organization

## References

[1] Dirlikov E, Raviglione M, Scano F (2015). Global tuberculosis control: toward the 2015 targets and beyond. Ann Intern Med.

[2] Glaziou P, Sismanidis C, Floyd K, Raviglione M (2015). Global epidemiology of tuberculosis. Cold Spring Harb Perspect Med.

[3] Raviglione MC (2006). The global plan to stop TB, 2006–2015. Int J Tuberc Lung Dis.

[4] Uplekar M, Weil D, Lonnroth K, Jaramillo E, Lienhardt C, Dias HM, Falzon D, Floyd K, Gargioni G, Getahun H (2015). WHO’s new end TB strategy. Lancet.

[5] WHO. Global tuberculosis report 2015. In: Geneva: World health Organization; 2015.

[6] McNerney R, Maeurer M, Abubakar I, Marais B, Mchugh TD, Ford N, Weyer K, Lawn S, Grobusch MP, Memish Z (2012). Tuberculosis diagnostics and biomarkers: needs, challenges, recent advances, and opportunities. J Infect Dis.

[7] Abubakar I, Lipman M, McHugh TD, Fletcher H (2016). Uniting to end the TB epidemic: advances in disease control from prevention to better diagnosis and treatment. BMC Med.

[8] Ranzani OT, Carvalho CRR, Waldman EA, Rodrigues LC (2016). The impact of being homeless on the unsuccessful outcome of treatment of pulmonary TB in São Paulo State, Brazil. BMC Med.

[9] WHO (2013). Global strategy and targets for tuberculosis prevention, care and control after 2015. Report by Secretariat. WHA65/2012/REC/3. 2013; Geneva.

[10] WHO (2014). Global tuberculosis report 2014. 2014.

[11] Murray CJL, Ortblad KF, Guinovart C, Lim SS, Wolock TM, Roberts DA, Dansereau EA, Graetz N, Barber RM, Brown JC (2014). Global, regional, and national incidence and mortality for HIV, tuberculosis, and malaria during 1990–2013: a systematic analysis for the Global Burden of Disease Study 2013. Lancet.

[12] Field N, Lim MS, Murray J, Dowdeswell RJ, Glynn JR, Sonnenberg P (2014). Timing, rates, and causes of death in a large South African tuberculosis programme. BMC Infect Dis.

[13] Jonnalagada S, Harries AD, Zachariah R, Satyanarayana S, Tetali S, Keshav Chander G, Rao S, Rao R, Peri S, Anchala R (2011). The timing of death in patients with tuberculosis who die during anti-tuberculosis treatment in Andhra Pradesh, South India. BMC Public Health.

[14] Waitt CJ, Peter K, Banda N, White SA, Kampmann B, Kumwenda J, Heyderman RS, Pirmohamed M, Squire SB (2011). Early deaths during tuberculosis treatment are associated with depressed innate responses, bacterial infection, and tuberculosis progression. J Infect Dis.

[15] Bisson GP, Zetola N, Collman RG (2015). Persistent high mortality in advanced HIV/TB despite appropriate antiretroviral and antitubercular therapy: an emerging challenge. Current HIV/AIDS Reports.

[16] Straetemans M, Glaziou P, Bierrenbach AL, Sismanidis C, van der Werf MJ (2011). Assessing tuberculosis case fatality ratio: a meta-analysis. PLoS One.

[17] WHO. Global tuberculosis report 2016. In: Geneva: World health Organization; 2016.

[18] National TB and Leprosy Control Programme. NTBLCP 2014 Annual Report. Abuja, Nigeria. 2014. http://www.health.gov.ng/doc/NTBLCP%202014%20Annual%20report-2.pdf. Accessed 11 Apr 2016.

[19] National Agency for the Control of AIDS. Global AIDS Response: Country Progress Report. Abuja. 2015. http://www.unaids.org/sites/default/files/country/documents/NGA_narrative_report_2015.pdf. Accessed 18 May 2016.

[20] Alobu I, Oshi SN, Oshi DC, Ukwaja KN (2014). Risk factors of treatment default and death among tuberculosis patients in a resource-limited setting. Asian Pac J Trop Med.

[21] Dauda MM (2010). Evaluation of the efficacy of directly observed treatment short course (DOTS) in patients with tuberculosis and HIV Co-infection in Kano, Nigeria. Rev Infect.

[22] Fatiregun AA, Ojo AS, Bamgboye AE (2009). Treatment outcomes among pulmonary tuberculosis patients at treatment centers in Ibadan, Nigeria. Ann Afr Med.

[23] Ifebunandu NA, Ukwaja KN, Obi SN (2012). Treatment outcome of HIV-associated tuberculosis in a resource-poor setting. Trop Dr.

[24] Ige OM, Akindele MO (2011). Five year review of treatment outcome of directly observed therapy (DOT) for re-treatment pulmonary tuberculosis patients in UCH, Ibadan, Nigeria. Afr J Med Med Sci.

[25] Ukwaja KN, Oshi DC, Oshi SN, Alobu I (2014). Profile and treatment outcome of smear-positive TB patients who failed to smear convert after 2 months of treatment in Nigeria. Trans R Soc Trop Med Hyg.

[26] Peters EJ, Ekott JU, Eshiet GA, Ayanechi CC (2005). Tuberculosis in Calabar: a ten-year review (1994–2003). Niger J Med.

[27] Salami AK, Oluboyo PO (2003). Management outcome of pulmonary tuberculosis: a nine year review in Ilorin. West Afr J Med.

[28] Halilu TB, Bala Z, Florence S (2013). Multi-drug resistance tuberculosis (mdr-tb) survey in North East Nigeria. J Pharm Cosmet Sci Vol.

[29] WHO (2013). Definitions and reporting framework for tuberculosis - 2013 revision (updated December 2014).

[30] Amoran OE (2013). Rates and risk factors for mortality among tuberculosis patients on directly observed therapy in rural primary health care centres in Ogun State, Nigeria. Niger Med Pract.

[31] Omotosho BA, Adebayo AM, Adeniyi BO, Ayodeji OO, Ilesanmi OS, Kareem AO, Akitikori OT, Erhabor GE (2014). Tuberculosis treatment outcomes and interruption among patients assessing DOTS regimen in a tertiary hospital in semi-urban area of south-western Nigeria. Niger J Med.

[32] Oshi DC, Oshi SN, Alobu I, Ukwaja KN (2014). Profile and treatment outcomes of tuberculosis in the elderly in Southeastern Nigeria, 2011–2012. PLoS One.

[33] Oladimeji O, Isaakidis P, Obasanya OJ, Eltayeb O, Khogali M, Van den Bergh R, Kumar AM, Hinderaker SG, Abdurrahman ST, Lawson L (2014). Intensive-phase treatment outcomes among hospitalized multidrug-resistant tuberculosis patients: results from a nationwide cohort in Nigeria. PLoS One.

[34] Corbett EL, Marston B, Churchyard GJ, De Cock KM (2006). Tuberculosis in sub-Saharan Africa: opportunities, challenges, and change in the era of antiretroviral treatment. Lancet.

[35] Yen YF, Yen MY, Shih HC, Hu BS, Ho BL, Li LH, Hsiao JC, Deng CY (2013). Prognostic factors associated with mortality before and during anti-tuberculosis treatment. Int J Tuberc Lung Dis.

[36] Lin Y-S, Yen Y-F (2015). Determinants of mortality before start of and during tuberculosis treatment among elderly patients: a population-based retrospective cohort study. Age Ageing..

[37] Low S, Ang LW, Cutter J, James L, Chee CBE, Wang YT, Chew SK (2009). Mortality among tuberculosis patients on treatment in Singapore. Int J Tuberc Lung Dis.

[38] Harries AD, Hargreaves NJ, Gausi F, Kwanjana JH, Salaniponi FM (2001). High early death rate in tuberculosis patients in Malawi. Int J Tuberc Lung Dis.

[39] Pepper DJ, Rebe K, Morroni C, Wilkinson RJ, Meintjes G (2009). Clinical deterioration during antitubercular treatment at a District Hospital in South Africa: the importance of drug resistance and AIDS defining illnesses. PLoS One.

[40] Greenberg AE, Lucas S, Tossou O, Coulibaly IM, Coulibaly D, Kassim S, Ackah A, De Cock KM (1995). Autopsy-proven causes of death in HIV-infected patients treated for tuberculosis in Abidjan, Cote d’Ivoire. Aids.

[41] Gandhi NR, Moll A, Sturm AW, Pawinski R, Govender T, Lalloo U, Zeller K, Andrews J, Friedland G (2006). Extensively drug-resistant tuberculosis as a cause of death in patients co-infected with tuberculosis and HIV in a rural area of South Africa. Lancet.

[42] Waitt CJ, Squire SB (2011). A systematic review of risk factors for death in adults during and after tuberculosis treatment. Int J Tuberc Lung Dis.

[43] Gupta A, Nadkarni G, Yang WT, Chandrasekhar A, Gupte N, Bisson GP, Hosseinipour M, Gummadi N (2011). Early mortality in adults initiating antiretroviral therapy (ART) in low- and middle-income countries (LMIC): a systematic review and meta-analysis. PLoS One.

[44] Mukadi YD, Maher D, Harries A (2001). Tuberculosis case fatality rates in high HIV prevalence populations in sub-Saharan Africa. Aids.

[45] Sreeramareddy CT, Qin ZZ, Satyanarayana S, Subbaraman R, Pai M (2014). Delays in diagnosis and treatment of pulmonary tuberculosis in India: a systematic review. Int J Tuberc Lung Dis.

[46] Cai J, Wang X, Ma A, Wang Q, Han X, Li Y (2015). Factors associated with patient and provider delays for tuberculosis diagnosis and treatment in Asia: a systematic review and meta-analysis. PLoS One.

[47] Lawn SD, Afful B, Acheampong JW (1998). Pulmonary tuberculosis: diagnostic delay in Ghanaian adults. Int J Tuberc Lung Dis.

[48] Needham DM, Foster SD, Tomlinson G, Godfrey-Faussett P (2001). Socio-economic, gender and health services factors affecting diagnostic delay for tuberculosis patients in urban Zambia. Trop Med Int Health.

[49] Hussen A, Biadgilign S, Tessema F, Mohammed S, Deribe K, Deribew A (2012). Treatment delay among pulmonary tuberculosis patients in pastoralist communities in Bale Zone, Southeast Ethiopia. BMC Res Notes.

[50] Kemp JR, Mann G, Simwaka BN, Salaniponi FM, Squire SB (2007). Can Malawi’s poor afford free tuberculosis services? Patient and household costs associated with a tuberculosis diagnosis in Lilongwe. Bull World Health Organ.

[51] Umar NA, Abubakar I, Fordham R, Bachmann M (2012). Direct costs of pulmonary tuberculosis among patients receiving treatment in Bauchi State, Nigeria. Int J Tuberc Lung Dis.

[52] Ukwaja KN, Alobu I, lgwenyi C, Hopewell PC (2013). The high cost of free tuberculosis services: patient and household costs associated with tuberculosis care in Ebonyi State, Nigeria. PLoS One.

[53] Kiwuwa MS, Charles K, Harriet MK (2005). Patient and health service delay in pulmonary tuberculosis patients attending a referral hospital: a cross-sectional study. BMC Public Health.

[54] Saifodine A, Gudo PS, Sidat M, Black J (2013). Patient and health system delay among patients with pulmonary tuberculosis in Beira city, Mozambique. BMC Public Health.

[55] Osei E, Akweongo P, Binka F (2015). Factors associated with DELAY in diagnosis among tuberculosis patients in Hohoe Municipality, Ghana. BMC Public Health.

[56] Virenfeldt J, Rudolf F, Camara C, Furtado A, Gomes V, Aaby P, Petersen E, Wejse C (2014). Treatment delay affects clinical severity of tuberculosis: a longitudinal cohort study. BMJ Open.

[57] Zerbini E, Chirico MC, Salvadores B, Amigot B, Estrada S, Algorry G (2008). Delay in tuberculosis diagnosis and treatment in four provinces of Argentina. Int J Tuberc Lung Dis.

[58] Lew W, Pai M, Oxlade O, Martin D, Menzies D (2008). Initial drug resistance and tuberculosis treatment outcomes: systematic review and meta-analysis. Ann Intern Med.

[59] Yuen CM, Amanullah F, Dharmadhikari A, Nardell EA, Seddon JA, Vasilyeva I, Zhao Y, Keshavjee S, Becerra MC (2015). Turning off the tap: stopping tuberculosis transmission through active case-finding and prompt effective treatment. Lancet (London, England).

[60] Adamu AU, Hafiz TR (2015). Multi-Drug Resistant Tuberculosis Pattern in Kano Metropolis. Nigeria. J Am Scie..

[61] Nglazi MD, Bekker LG, Wood R, Kaplan R (2015). The impact of HIV status and antiretroviral treatment on TB treatment outcomes of new tuberculosis patients attending co-located TB and ART services in South Africa: a retrospective cohort study. BMC Infect Dis.

[62] Koenig SP, Riviere C, Leger P, Joseph P, Severe P, Parker K, Collins S, Lee E, Pape JW, Fitzgerald DW (2009). High mortality among patients with AIDS Who received a diagnosis of tuberculosis in the first 3 months of antiretroviral therapy. Clin Infect Dis.

[63] Moore D, Liechty C, Ekwaru P, Were W, Mwima G, Solberg P, Rutherford G, Mermin J (2007). Prevalence, incidence and mortality associated with tuberculosis in HIV-infected patients initiating antiretroviral therapy in rural Uganda. Aids.

[64] Abdool Karim SS, Naidoo K, Grobler A, Padayatchi N, Baxter C, Gray A, Gengiah T, Nair G, Bamber S, Singh A (2010). Timing of initiation of antiretroviral drugs during tuberculosis therapy. N Engl J Med.

[65] Pepper DJ, Schomaker M, Wilkinson RJ, Azevedo V, Maartens G (2015). Independent predictors of tuberculosis mortality in a high HIV prevalence setting: a retrospective cohort study. AIDS Res Ther.

[66] Saraceni V, Durovni B, Cavalcante SC, Cohn S, Pacheco AG, Moulton LH, Chaisson RE, Golub JE (2014). Survival of HIV patients with tuberculosis started on simultaneous or deferred HAART in the THRio cohort, Rio de Janeiro, Brazil. Braz J Infect Dis.

[67] Yates TA, Khan PY, Knight GM, Taylor JG, McHugh TD, Lipman M, White RG, Cohen T, Cobelens FG, Wood R (2016). The transmission of Mycobacterium tuberculosis in high burden settings. Lancet Infect Dis.

[68] Macpherson P, Dimairo M, Bandason T, Zezai A, Munyati SS, Butterworth AE, Mungofa S, Rusakaniko S, Fielding K, Mason PR (2012). Risk factors for mortality in smear-negative tuberculosis suspects: a cohort study in Harare, Zimbabwe. Int J Tuberc Lung Dis.

[69] Cain KP, Anekthananon T, Burapat C, Akksilp S, Mankhatitham W, Srinak C, Nateniyom S, Sattayawuthipong W, Tasaneeyapan T, Varma JK (2009). Causes of death in HIV-infected persons who have tuberculosis, Thailand. Emerg Infect Dis.

[70] Pedrazzoli D, Abubakar I, Potts H, Hunter PR, Kruijshaar ME, Kon OM, Southern J (2015). Risk factors for the misdiagnosis of tuberculosis in the UK, 2001–2011. Eur Respir J.

[71] Field N, Murray J, Wong ML, Dowdeswell R, Dudumayo N, Rametsi L, Martinson N, Lipman M, Glynn JR, Sonnenberg P (2011). Missed opportunities in TB diagnosis: a TB Process-Based Performance Review tool to evaluate and improve clinical care. BMC Public Health.

[72] Sonnenberg P, Lim MS, Dowdeswell RJ, Field N, Glynn JR, Murray J (2012). Quantifying errors in the estimation of tuberculosis mortality in a population of South African miners. Int J Tuberc Lung Dis.

[73] Squire SB, Belaye AK, Kashoti A, Salaniponi FML, Mundy CJF, Theobald S, Kemp J (2005). ‘Lost’ smear-positive pulmonary tuberculosis cases: where are they and why did we lose them?. Int J Tuberc Lung Dis.

